# Survey dataset on analysis of queues in some selected banks in Ogun State, Nigeria

**DOI:** 10.1016/j.dib.2018.05.101

**Published:** 2018-05-24

**Authors:** Sheila A. Bishop, Hilary I. Okagbue, Pelumi E. Oguntunde, Abiodun A. Opanuga, Oluwole A. Odetunmibi

**Affiliations:** Department of Mathematics, Covenant University Canaanland, Ota, Nigeria

**Keywords:** Queues, Banks, Waiting time, Service, Length, Urban areas, Statistics

## Abstract

Queuing theory is the mathematical study of waiting queues (or lines). The theory enables the mathematical analysis of several related processes such as arriving at the queue, waiting in line and being served by a server. This data article contains the analysis of queuing systems obtained from queues from the observed data of some selected banks in Ogun State. One of the gains expected from this survey, is to help review the efficiency of the models used by banks in such geographical locations in sub-Saharan countries. The Survey attempts to estimate the average waiting time and length of queue(s).

**Specifications Table**

**Table:** 

Subject area	Decision sciences
More specific subject area	Queuing analysis, operations research, statistics
Type of data	Tables
How data was acquired	Field Survey and with the aid of stop watch and a recorder.
Data format	Analyzed
Experimental factors	Simple random sampling of some selected Banks in Urban areas of Ogun State, Nigeria.
Experimental features	Analysis of the waiting and service times of selected customers.
Data source location	Covenant University Ota, Ogun State, Nigeria
Data accessibility	All the data are in this data article

**Value of the data**

• The data could be useful in detecting the causes and proffering solutions to the problem of queues.
• Queues are necessary if order is to be maintained in the society, but most queues in sub-Saharan countries constitute a menace and sometimes end in riot and mob actions. Hence the data can be useful for security agents responsible for maintaining law and order [1], [2].
• The data could be used by banking regulatory bodies in Nigeria.
• The analysis of the data could be helpful in time management especially at peak periods [3].
• The data can also help the banks to improve on their services [4], [5], [6].
• The data can also help to rate the banks in terms of customers services satisfaction.

## 1 Data

The data was collected from three banks in three different urban areas of Ogun State. The Data was generated using a stop watch and a recorder to note the arrival time, the time spent on the queue (waiting time) before being attended to and the time used to serve a customer (Service time).

The notations used for the presentation of data are **X**_**1**_**, X**_**2**_**, X**_**3**_**,** and **N**_**1**_ for the first bank **Y**_**1**_**, Y**_**2**_**, Y**_**3**_**,** and **N**_**2**_ for the second bank and **Z**_**1**_**, Z**_**2**_**, Z**_**3**_**,** and **N**_**3**_ for the third bank respectively. They denote the following:

**X**_**1**_**, Y**_**1**_ and **Z**_**1**_ represents the time range when a customer arrives at the bank and the time his/her cheque or withdrawal booklet was collected for the first, second and third bank respectively.

**X**_**2**_**, Y**_**2**_ and **Z**_**2**_ represents the time used to process the cheque or withdrawal booklet in the first, second, and third banks respectively.

**X**_**3**_**, Y**_**3**_ and **Z**_**3**_ represents the total time in the system in the first, second, and third banks respectively.

**N**_**1**_**, N**_**2**_ and **N**_**3**_ represents the number of people who came to the first, second and third banks and were attended to.

The data taken covers only twelve weeks. Four weeks for each bank and the time is measured in minutes.

## 2 Experimental design, materials and methods

The study of queues is the study of waiting times which often results to models that predicts queue length and waiting time. The models are also used to make decisions on how to increase servers, optimize queue length and waiting time. Queue is often characterized by the following presented in Table 1.

**Table 1:** Features of queue.

1	Queue is a linear data structure.
2	In queues insertion can take place at only one end called rear.
3	In queues deletions can takes place at the other end called front.
4	Queues are called FIFO (first in first out). The element first into the queue is the element deleted first from the queue.
5	Queues are also called LILO (last in last out).The element entered last into the queue is the element deleted last from the queue.

Several operations can be done on queues which are listed as:

1. **Insertion:** inserting a new element into the queue.
2. **Deletion:** deleting a new element from the queue.
3. **Display:** visit each node at least once.

• Queue is full- there is no room to insert a new element.
• Queue is empty- there is no element to delete from queue.

There are several methods of investigating phenomena that are modeled as queuing problems. Some are mentioned as follows:

i. Direct observation of practical situation
ii. The planned experiment under artificial conditions
iii. The simulation method
iv. The Mathematical Analysis method
v. Product-form solutions method
vi. Methods from complex-function theory
vii. Analytic-algorithmic methods
viii. Heavy and light traffic approximations

It is noteworthy that not all queuing problems can be investigated mathematically. Some investigators using (i) and (ii) above require a clear out study of the situation and therefore, necessary adjustments and manipulations are made.

### 2.1 Method of data collection

The investigators made use of (i) and (ii) mentioned above and with the aid of a stop watch and a recorder.

### 2.2 Data presentation

The data are presented in Table 2, Table 3, Table 4. It should be noted that the departure time was not captured because the customers often wait behind to count their money, wait for those that accompanied them or make non-transaction activities such as renewal of Automated teller machine (ATM) cards, registration of bank verification number, enquiries on new banking products and other complaints. The raw data containing the arrival times of the customers can be assessed as Supplementary Data.

**Table 2:** The queuing data for the first bank.

**Weeks**	**Days**	**X**_**1**_	**X**_**2**_	**X**_**3**_	**N**_**1**_
1st	MONDAY	12	26	38	880
	TUESDAY	5	19	24	720
	WEDNESDAY	6	8	14	1020
	THURSDAY	11	20	31	802
	FRIDAY	17	15	32	522
	MONDAY	20	13	33	989
	TUESDAY	22	18	40	684
2nd	WEDNESDAY	24	19	43	548
	THURSDAY	23	9	32	1021
	FRIDAY	25	20	45	789
	MONDAY	8	15	23	1000
	TUESDAY	10	22	32	990
3rd	WEDNESDAY	11	10	21	1001
	THURSDAY	10	15	25	1051
	FRIDAY	7	17	24	982
	MONDAY	7	9	16	857
	TUESDAY	10	9	19	981
4th	WEDNESDAY	10	6	16	1057
	THURSDAY	5	20	25	899
	FRIDAY	10	12	22	996
	**Total**	**253**	**302**	555	17,789

**Table 3:** The queuing data for the second bank.

**Weeks**	**Days**	**Y**_**1**_	**Y**_**2**_	**Y**_**3**_	**N**_**2**_
1st	MONDAY	16	8	24	1034
	TUESDAY	17	15	32	789
	WEDNESDAY	18	8	26	1002
	THURSDAY	13	15	28	910
	FRIDAY	10	6	16	931
	MONDAY	16	14	30	748
	TUESDAY	14	9	23	924
2nd	WEDNESDAY	9	17	26	872
	THURSDAY	18	10	28	764
	FRIDAY	15	10	25	890
	MONDAY	15	19	34	971
	TUESDAY	23	18	41	685
3rd	WEDNESDAY	30	10	40	724
	THURSDAY	28	9	37	873
	FRIDAY	26	18	44	605
	MONDAY	10	32	42	1017
	TUESDAY	7	17	24	1009
4th	WEDNESDAY	12	19	31	891
	THURSDAY	11	26	37	948
	FRIDAY	13	14	27	901
	**Total**	**321**	**294**	**615**	17,488

**Table 4:** The queuing data for the third bank.

**Weeks**	**Days**	**Z**_**1**_	**Z**_**2**_	**Z**_**3**_	**N**_**3**_
1st	MONDAY	10	12	22	767
	TUESDAY	12	11	23	930
	WEDNESDAY	7	7	14	921
	THURSDAY	22	10	32	878
	FRIDAY	11	12	23	790
	MONDAY	11	18	29	876
	TUESDAY	18	14	32	923
2nd	WEDNESDAY	12	14	26	910
	THURSDAY	10	18	28	1002
	FRIDAY	9	8	17	949
	MONDAY	16	10	26	934
	TUESDAY	8	6	14	1011
3rd	WEDNESDAY	12	7	19	874
	THURSDAY	8	10	18	762
	FRIDAY	6	9	15	631
	MONDAY	13	12	25	989
	TUESDAY	15	8	23	784
4th	WEDNESDAY	16	14	30	648
	THURSDAY	10	8	18	891
	FRIDAY	11	15	26	752
	**Total**	**237**	**223**	460	17,222

### 2.3 Descriptive statistics

The descriptive statistics for the data are summarized as follows for the data of the first, second and the third banks respectively. These are shown in Table 5, Table 6, Table 7.

**Table 5:** Description statistics for the queuing data of the first bank.

Statistic	**X**_**1**_	**X**_**2**_	**X**_**3**_	**N**_**1**_
Mean	12.65	15.1	27.75	889.45
Standard Error	1.483728	1.220224311	2.028578709	36.38272255
Median	10	15	25	981.5
Mode	10	20	32	#N/A
Standard Deviation	6.635431	5.457009013	9.072079782	162.7084816
Sample Variance	44.02895	29.77894737	82.30263158	26474.05
Kurtosis	−0.78814	−0.85103544	−0.74558109	0.318384698
Skewness	0.800322	0.058384533	0.371099032	−1.13689222
Range	20	20	31	535
Minimum	5	6	14	522
Maximum	25	26	45	1057

**Table 6:** Description statistics for the queuing data of the second bank.

Statistic	**Y**_**1**_	**Y**_**2**_	**Y**_**3**_	**N**_**2**_
Mean	16.05	14.4	30.75	874.4
Standard Error	1.418812	1.350244	1.669975	26.63826
Median	15	14.5	29	896
Mode	16	10	24	#N/A
Standard Deviation	6.345118	6.038473	7.468354	119.1299
Sample Variance	40.26053	36.46316	55.77632	14191.94
Kurtosis	0.177576	2.405767	−0.61616	−0.19879
Skewness	0.906584	1.132334	0.190103	−0.71182
Range	23	26	28	429
Minimum	7	6	16	605
Maximum	30	32	44	1034

**Table 7:** Description statistics for the queuing data of the third bank.

Statistic	**Z**_**1**_	**Z**_**2**_	**Z**_**3**_	**N**_**3**_
Mean	11.85	11.15	23	861.1
Standard Error	0.880416	0.785644	1.289635	24.48328
Median	11	10.5	23	884.5
Mode	10	12	23	#N/A
Standard Deviation	3.937338	3.513508	5.767422	109.4926
Sample Variance	15.50263	12.34474	33.26316	11988.62
Kurtosis	0.97078	−0.48582	−1.07621	−0.24494
Skewness	0.940453	0.516912	−0.0823	−0.69166
Range	16	12	18	380
Minimum	6	6	14	631
Maximum	22	18	32	1011

### 2.4 Analysis of variance

Analysis of variance (ANOVA) is done to investigate mean differences among the total time spent by the customers in the three banks. The result is presented in Table 8.

**Table 8:** ANOVA result.

*Source of Variation*	*SS*	*df*	*MS*	*F*	*P-value*	*F crit*
Between Groups	610.8333	2	305.4167	5.347489	0.00744	3.158843
Within Groups	3255.5	57	57.11404			
Total	3866.333	59				

There are significant mean differences among the total time spent by the customers in the three banks at 0.05 level of significance.

Further analysis of data can be carried out in the following areas using any of the statistical tools applied in Refs. [7], [8], [9], [10], [11].

i. The utilization factor or traffic intensity can be calculated using the arrival rate and the service time. This can used to determine average needed servers, number of automated banking machines (ATM). See Refs. [2], [4], [5], [6].
ii. The confidence intervals for average service rate and average arrival rate can be estimated assuming the service time and arrival time are independent and identically distributed.
iii. The data can be analyzed pictorially, that is using a Bar chat, Pie chat to show the traffic intensity and efficiency of the servers.
iv. The results from each bank can be compared to determine the level of service efficiency.
