# Supplementary material for: Survey dataset on analysis of queues in some selected banks in Ogun State, Nigeria
Source: Data Brief. 2018 May 24;19:835–41. doi: 10.1016/j.dib.2018.05.101 (PMC5997939; doi:10.1016/j.dib.2018.05.101)
Supplement: Supplementary file 1 — Supplementary material [file mmc1.docx]

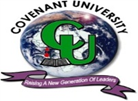


**COVENANT UNIVERSITY, OTA NIGERIA**

**COLLEGE OF SCIENCE AND TECHNOLOGY**

**MATHEMATICS DEPARTMENT**

**CONFLICT OF INTEREST**

All the authors made some valuable contributions and state that there are no competing interests in the publication of this research work

Thank you

Sheila A. Bishop

14^th^ May, 2018
